# Dramatic Remodeling of the Gut Microbiome Around Parturition and Its Relationship With Host Serum Metabolic Changes in Sows

**DOI:** 10.3389/fmicb.2019.02123

**Published:** 2019-09-12

**Authors:** Xiaochang Huang, Jun Gao, Yuanzhang Zhao, Maozhang He, Shanlin Ke, Jinyuan Wu, Yunyan Zhou, Hao Fu, Hui Yang, Congying Chen, Lusheng Huang

**Affiliations:** State Key Laboratory of Pig Genetic Improvement and Production Technology, Jiangxi Agricultural University, Nanchang, China

**Keywords:** gut microbiome, phylogenetic composition, metabolomics, perinatal period, sow

## Abstract

Perinatal care is important in mammals due to its contribution to fetal growth, maternal health, and lactation. Substantial changes in host hormones, metabolism, and immunity around the parturition period may be accompanied by alterations in the gut microbiome. However, to our knowledge, changes in the gut microbiome and their contribution to the shifts in host metabolism around parturition have not been investigated in pigs. Furthermore, pigs are an ideal biomedical model for studying the interactions of the gut microbiota with host metabolism, due to the ease of controlling feeding conditions. Here we report dramatic remodeling of the gut microbiota and the potential functional capacity during the late stages of pregnancy (5 days before parturition, LP) to postpartum (within 6 h after delivery, PO) in both experimental and validated populations of sows (*n* = 107). The richness of bacteria in the gut of both pregnant and delivery sows significantly decreased, whilst the β-diversity dramatically expanded. The ratio of Bacteroidetes to Firmicutes, and the relative abundance of Prevotella significantly decreased, whilst the relative abundance of the predominant genus *Lactobacillus* significantly increased from LP to PO state. The predicted functional capacities of the gut microbiome related to amino acid metabolism, the metabolism of cofactors and vitamins, and glycan biosynthesis were significantly decreased from LP to PO state. However, the abundance of the functional capacities associated with carbohydrate and lipid metabolism were increased. Consistent with these changes, serum metabolites enriched at the LP stage were associated with the metabolism of amino acids and vitamins. In contrast, metabolites enriched at the PO stage were related to lipid metabolism. We further identified that the richness and β-diversity of the gut microbiota and the abundance of *Lactobacillus* accounted for shifts in the levels of bile acid metabolites associated with lipid metabolism. The results suggest that host-microbiota interactions during the perinatal period impact host metabolism. These benefit the lactation of sows by providing energy from lipid metabolism for milk production.

## Introduction

Perinatal period (from 7 days before delivery to 7 days postpartum) is a unique and critical time during the reproduction cycle of sows. Correct feeding and management during this period have profound effects on the production levels of both sows and piglets. Approximately 60% of fetal growth or uterine energy deposition occurs during the final 30 days of pregnancy in pigs (Noblet et al., 1990). The withdrawal of progesterone prior to parturition triggers a succession of hormonal changes that lead to farrowing (Liptrap, 1980). Perinatal care is also important in women due to its relationship to puerperal health and infant growth. The physiological state of females during the perinatal period (including hormone levels, metabolism, and immunity) undergoes dramatic changes (Newbern and Freemark, 2011). In the metabolic state as the perinatal period approaches, insulin sensitivity is reduced, but insulin resistance gradually increases (Barbour et al., 2007). Furthermore, each stage of pregnancy and parturition is faced with an array of immunological challenges (Mor and Cardenas, 2010). Upon the completion of fetal development, pro-inflammatory nuclear factor-κB (NF-κB) signaling initiates labor and plays a crucial role during labor and delivery (Lappas and Rice, 2007).

Neuman and Koren suggested that hormonal levels are likely to influence the microbial composition of the gut during pregnancy since hormones influence bacterial growth (Neuman and Koren, 2017). Previous studies have indicated that reduced insulin sensitivity and increased insulin resistance are related to the gut microbiota (Vijay-Kumar et al., 2010). The immune system also profoundly influences the composition of gut microbiota (Salzman et al., 2010), whilst gut microbiota inhibit NF-κB activation (Lim and Kim, 2017). Based on these observations, substantial changes in host hormones, metabolism, and immunity around parturition may accompany alterations of the gut microbiota. Previous studies have indicated that gut microbiota undergoes a significant shift during pregnancy (Santacruz et al., 2010; Koren et al., 2012), particularly during the third trimester. Gut microbiota from the period of non-pregnancy to pregnancy is characterized with the reduced richness and the increased between-subject diversity. The relative abundance of *Actinobacteria* and *Proteobacteria* increases on average from the first to the third trimester of pregnancy (Koren et al., 2012). Gut microbiota at the third trimester induce greater adiposity and insulin insensitivity than the microbiota during the first trimester (Koren et al., 2012). Studies by Santacruz et al. indicated that gut microbiota composition is associated with body weight, weight gain and biochemical parameters in pregnant women (Santacruz et al., 2010). However, to our knowledge, changes in the phylogenetic composition and functional capacity of the gut microbiome during the perinatal period have not been reported, and the contribution of gut microbiota to changes in host metabolism during the perinatal period therefore remains undefined in pigs.

Pigs provide an ideal biomedical model for studying the relationship between the gut microbiome and host metabolism, due to the ease of control of their feeding conditions. In this study, we characterized the shifts in the phylogenetic composition and potential functional capacity of the gut microbiome occurring from late pregnancy (~5 days before parturition, defined as the LP state) to postpartum (within 6 h after parturition, defined as the PO state). We observed profound alterations in the gut microbial community structure and the potential functional capacity, evidenced in two sow populations. The metabolic profiles of serum metabolites of the experimental sows were also measured. We identified a significant correlation between alterations of the gut microbiota and shifts in host serum metabolites from LP to PO, suggesting that the contribution of gut microbiota to changes in host metabolism occur during the perinatal period.

## Materials and Methods

### Experimental Animals and Sample Collection

All experimental sows were from a heterogeneous cross population, produced by random hybridization amongst four Western (Pietrain, Duroc, Landrace, and Large White) and Chinese pig breeds (Bamaxiang, Erhualian, Laiwu, and Zang). Feces samples from 45 sows which were used to generate F_6_ pigs and hereinafter referred to as F_5_ sows (as tested samples) and 62 sows used to reproduce F_7_ pigs and hereinafter referred to as F_6_ sows (as validated samples) were collected at 5 days prior to the predicted parturition date and 6 h following parturition. Feces samples from the other 150 non-pregnant F_6_ sows of the same heterogeneous cross population were used as the study controls. All sows were raised in independent pens and provided the same formula feed twice daily containing 16% crude protein, 3,100 kj digestible energy and 0.78% lysine. Water was available *ad libitum* from nipple drinkers. All sows were healthy and received no probiotic or antibiotic therapy within two months of sample collection. Fecal samples were immediately dipped in liquid nitrogen for transportation, and stored at −80°C until use. Blood was harvested from 22 of the 62 F_6_ sows at the two time points. Serum samples were centrifuged and stored at −80°C prior to analysis.

### Ethics Statement

All animal procedures were conducted according to the guidelines for the care and use of experimental animals established by the Ministry of Agriculture of China. The project was approved by Animal Care and Use Committee (ACUC) in Jiangxi Agricultural University (No. JXAU2011-006).

### Microbial DNA Extraction of Fecal Samples and 16S rRNA Gene Sequencing

QIAamp Fast DNA Stool Mini Kit (Qiagen, Germany) was used to extract microbial DNA from the fecal samples according to the manual protocol. The concentration and quality of DNA were determined by 0.8% agarose gel electrophoresis and on a Nanodrop-1000 (Thermo Scientific, USA). The V3 - V4 hypervariable region of the 16S rRNA gene was amplified with the barcode fusion primers (338F: 5-ACTCCTACGGGAGGCAGCAG-3, 806R: 5-GGACTACHVGGGTWTCTAAT-3). Following purification, PCR products were used for library construction and sequenced on an Illumina MiSeq platform (Illumina, USA). The 16S rRNA gene sequencing data were submitted to the SRA database in NCBI with the accession number PRJNA518709. Barcode sequences and low-quality reads were filtered to obtain clean reads. Pair-end clean sequences were then merged into tags using FLASH software (Magoc and Salzberg, 2011). To obtain microbial taxonomy and abundance data, tags were clustered into operational taxonomic unit (OTUs) at 97% similarity after chimeras were removed using VSEARCH (Rognes et al., 2016). Representative sequences of each OTU were then matched to the RDP reference database to obtain the microbial taxonomy information (Desantis et al., 2006). To avoid the influence of sequencing depth during statistical analysis, we limited the library size to 10,000 tags for all samples.

### Metabolomic Profiling of Sow Serum Samples

All serum samples were thawed on ice. A quality control (QC) sample produced by mixing and blending equal volumes of each tested serum sample were set for each of the 12 samples to estimate a mean profile of the analytes assessed during analysis. For this, 100 μl of serum was mixed with 300 μl of precooled methanol and incubated at −20°C for 3 h. Mixed samples were centrifuged for 15 min at a centrifugal speed of 15,000 rpm, and supernatants were collected and transferred to speedvac for crystallization. For metabolomic profiling, concentrated products were resuspended in 150 μL of 15% methanol and transferred to a UPLC-QTOFMS system (Waters Corp., USA) for data collection.

Treated samples were injected into 2.1 × 100 mm Acquity UPLCTM BEH C18 columns packed with 1.7 μm particles and held at 40 °C under the UPLC system. The solvent system included 1% acetonitrile and 0.1% formic acid in the gradient elution mode for both electrospray positive (ES+) and negative ion mode (ES–) analyses under a constant temperature of 8 °C. Mass spectrometry data were collected using Waters Q-TOF Premier equipped with an electrospray source operating in either ES+ or ES–. Mass scanning was set to range of 50–1,200 m/z with a scan time of 0.3 s and interscan delay of 0.02 s over a 26- and 18-min analysis time, respectively. MassLynx software (Waters Corp., USA) was used for system control and data acquisition. Leucine enkephalin was used as the lock mass (m/z 556.2771 in ES+ and 554.2615 in ES–) at a concentration of 100 ng/mL, under a flow rate of 5 μL/min for all analyses.

Raw data from UPLC–QTOFMS underwent peak selection and grouping, retention time correction, second peak grouping, and isotope and adducts annotation using Progenesis QI (Rusilowicz, 2016). Each retained peak was then normalized to the QC sample using MetNormalize (Shen et al., 2016). The relative RSD value of the metabolites in the QC samples was set at a threshold of 30% to standardize the reproducibility of the metabolomic data sets.

For the annotation of serum metabolites, the HMDB database (Wishart et al., 2017) was used to align the molecular mass data (m/z) to identify relevant metabolites. If the differences between the observed and theoretical mass were ≤10 ppm, the metabolite was annotated to the mass. The molecular formula of the matched metabolites was further validated by isotopic distribution measurements and fragmentation similarity.

### Statistical Analysis

#### Comparison of the Microbial Composition and Potential Function Capacity of the Gut Microbiome Between Sows at Different Reproduction States

The α-diversity of fecal microbiota was calculated using Mothur (Schloss et al., 2009). The β-diversity of fecal microbiota was analyzed by QIIME (Kuczynski et al., 2011). Potential function capacities were predicted on PICRUSt software using the 16S rRNA sequencing data (Langille et al., 2013). To evaluate alterations of sow gut microbiota from LP to PO state, we first performed a PCoA analysis based on the OTU composition using the vegan package (Dixon, 2003). Comparisons of the α- and β-diversity indexes (observed species and within-group weighted Unifrac distance) between sows at different reproduction states were performed using T-tests. Because fecal samples were collected from the experimental sows at two timepoints, a Wilcoxon rank sum test (paired) was used to identify the bacterial taxa and predicted functional capacities showing significant differences in the relative abundance between LP and PO in both F_5_ and F_6_ sow populations (MacFarland and Yates, 2016). The significant threshold was set at an FDR < 0.05. As the non-pregnant F_6_ sows (BS) had fecal samples collected at only a single timepoint, non-paired Wilcoxon rank sum test were used to identify the differential microbial taxa and KEGG function terms amongst LP, PO and BS states in the F_6_ sows.

### Construction of the Serum Metabolite Modules and Enrichment Analysis of Differential Metabolite Features

We firstly corrected the effect of sample batches on serum metabolite profiles obtained from both the positive and negative ion mode using ComBat methods (Stein et al., 2015). We then constructed the co-abundant topological network of serum metabolites using the WGCNA package in R software with a scale-free topology criterion soft threshold of β = 5 (positive ion mode) and β = 4 (negative ion mode) (Langfelder and Horvath, 2008). Metabolic modules were isolated from topological networks using the dynamic hybrid tree-cutting algorithm with a minimum cluster size of five. The PC1 value of the metabolic module was summarized as the profile of each module. Similar modules were subsequently merged if the correlation between the eigen vectors of the serum metabolite clusters exceeded 0.8. Paired Wilcoxon rank sum tests were performed to identify the metabolic modules that significantly shifted from LP to PO at a significant threshold of FDR < 0.05. All metabolite features in the modules showing significant shifts from the LP to PO state were matched to the KEGG database to perform an enrichment analysis using methods of the untargeted metabolomic pathway analysis introduced into online metaboAnalyst 4.0 (Xia et al., 2015).

#### Correlation Analysis Between the Shifts of the Gut Microbiome and the Changes in Host Serum Metabolites

A total of 22 pairs of samples (22 sows were collected the samples at both LP and PO state) with both serum metabolome and gut microbiome data were used for evaluating the correlation between the shifts of the gut microbiome and the changes in host serum metabolites. We first calculated the abundance change for bacterial taxa and the PC1 value change for each metabolic module. A Spearman rank test was performed to evaluate the correlation between the shifts of bacterial taxa and serum metabolic modules at an FDR < 0.05. To identify the hubs of metabolite clusters associated with the shifts in bacterial abundance or the change of microbial diversity, metabolite interaction networks were constructed and visualized using cytoscape (v3.30) (Shannon et al., 2003).

## Results

### Remodeling of the Gut Microbiota From Late Pregnancy to Postpartum in Sows

**Figure 1:**
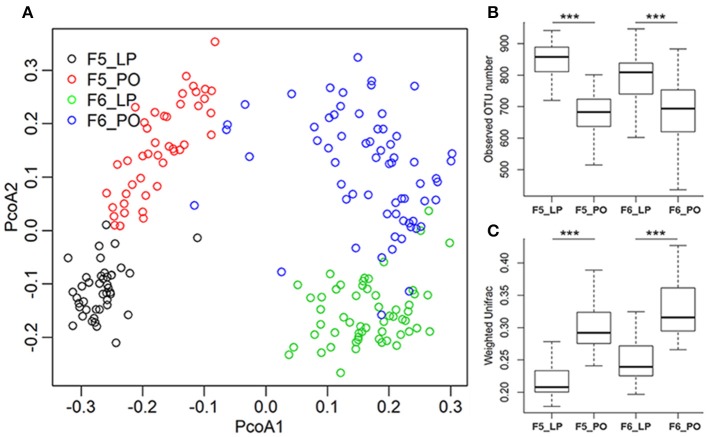
Remodeling of gut microbial composition in sows from later pregnancy to postpartum state. **(A)** PCoA analysis shows the different gut microbial compositions between sows at later pregnancy (LP) and postpartum (PO) state in both F_5_ and F_6_ sows. **(B,C)** Indicate the changes of gut microbial diversity from LP to PO state. **(B)** Observed species; **(C)** within-group weighted UniFrac distance (β-diversity). The *X*-axis shows the sample groups, and the *Y*-axis indicates observed species or the value of within-group weighted UniFrac distance. ***Represents the significance threshold of *P* < 0.005.

**Figure 2:**
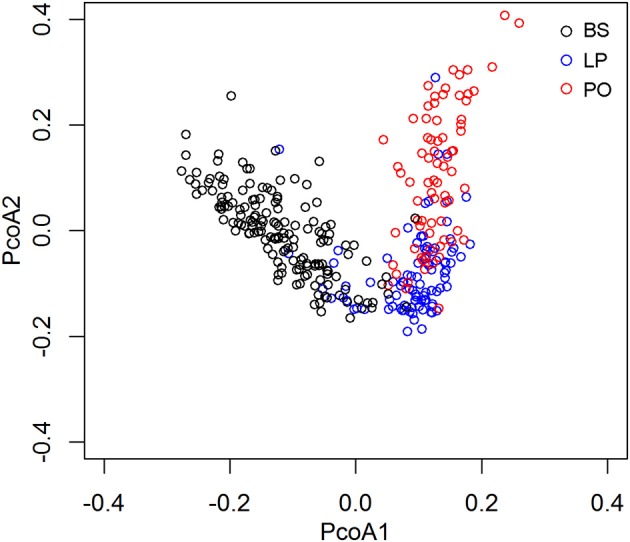
PCoA analysis based on weighted UniFrac shows the different gut microbial compositions among sows at non-pregnancy, later pregnancy, and postpartum state.

To evaluate alterations in sow gut microbiota from LP to PO, 90 fecal samples from 5 F_5_ sows were used for microbial composition analysis by 16 rRNA gene sequencing. We rarified the library size to 10,000 tags per sample. At a 97% pairwise sequence identity, an average of 760 operational taxonomic units (OTUs) per sample was obtained. PCA analysis identified distinct microbial diversity and composition between the LP and PO states (**Figure 2**). We compared the α- and β-diversity of gut microbial communities and identified significant reductions in OTU numbers at the PO stage (Figure 1B). Weighted Unifrac analysis revealed a dramatic expansion of β-diversity after parturition (Figure 1C). At the taxonomy level, we identified 10 phyla with distinct differences in their relative abundance between LP and PO. The relative abundance of *Firmicutes* (LP: 43.20 ± 6.35% vs. PO: 61.89 ± 9.03%, *P* = 7.62 × 10^−8^) and *Fusobacteria* (LP: 0.01 ± 0.04% vs. PO: 0.14 ± 0.39%, *P* = 3.90 × 10^−4^) increased on average from the LP to the PO state, whilst the relative abundance of the other eight phyla decreased from the LP to the PO state (Table S1), including *Bacteroidetes* which showed the most significant change in relative abundance (LP: 35.09 ± 5.28% vs. PO: 21.54 ± 5.04%, *P* = 3.41 × 10^−11^). At the genus level, we identified 43 genera with significant differences in their relative abundance from LP to PO, including 26 genera with a higher abundance at LP, and 17 genera with an increased abundance at PO. The relative abundance of *Lactobacillus* mostly increased (LP: 2.5 ± 2.15% vs. PO: 13.79 ± 8.86%, *P* = 3.50 × 10^−11^) from LP to PO, whilst the abundance of *SMB53* significantly decreased (LP: 0.4 ± 0.23% vs. PO: 0.15 ± 0.18%, *P* = 3.68 × 10^−7^). The abundance of the other predominant genus *Prevotella* decreased from 0.34 ± 4.55% (LP) to 4.59 ± 2.88% (PO, *P* = 1.96 × 10^−5^) (Table S1).

To confirm the distinct changes in the microbial community structure of sow feces from LP to PO, we determined the phylogenetic composition of fecal microbiota in a further 124 samples from 2 F_6_ sows of the same heterozygote population using the methods described above. An average of 747 OTUs were obtained per sample. The α- and β-diversity analyses of the F_5_ fecal microbial communities were reproduced in the F_6_ sows (Figure 1). At the taxonomy level, we identified 11 phyla and 40 genera showing significant differences in the relative abundance from LP to PO in F_6_ sows (Table S2). Six of the 11 phyla displayed similar changes to those of F_5_ sows, including two predominant phyla *Bacteroidetes* and *Firmicutes* (Table 1). At the genus level, the relative abundance of the 21 genera showed similarly significant changes to those of F_5_ sows, including 10 genera with a decreased abundance from LP to PO and 11 genera with an increased average abundance. The abundancy shifts in predominant bacterial genera from the LP to the PO state in F_6_ sows were highly comparable to those of F_5_ sows (Table 1).

**Table 1:** The bacterial taxa showing significant alteration of relative abundances from late pregnancy to postpartum in both F_5_ and F_6_ sows.

**Group**	**F_**5**__LP**	**F_**5**__PO**	**F_**6**__LP**	**F_**6**__PO**	**Shift direction**
**PHYLUM**					
*Bacteroidetes*	35.63 ± 7.03	22.09 ± 5.63	27.97 ± 7.43	19.13 ± 8.45	↓
*Firmicutes*	42.79 ± 6.98	61.49 ± 9.01	59.01 ± 8.73	69.67 ± 9.81	↑
*Verrucomicrobia*	3.42 ± 1.59	0.77 ± 0.91	0.66 ± 0.71	0.15 ± 0.23	↓
*Cyanobacteria*	0.66 ± 0.62	0.15 ± 0.14	0.46 ± 0.36	0.21 ± 0.24	↓
*Fusobacteria*	0.01 ± 0.03	0.13 ± 0.39	0.04 ± 0.23	0.23 ± 0.47	↑
*Fibrobacteres*	0.52 ± 0.49	0.41 ± 0.69	0.37 ± 0.67	0.14 ± 0.59	↓
**GENUS**					
*Lactobacillus*	2.23 ± 0.69	13.81 ± 8.95	6.67 ± 5.91	17.20 ± 14.41	↑
*Eubacterium*	0.05 ± 0.06	0.31 ± 0.43	0.04 ± 0.06	0.42 ± 0.39	↑
*Bulleidia*	0.04 ± 0.06	0.20 ± 0.37	0.07 ± 0.08	0.23 ± 0.28	↑
*Blautia*	0.34 ± 0.19	0.59 ± 0.44	0.20 ± 0.17	0.47 ± 0.36	↑
*Dorea*	0.21 ± 0.13	0.37 ± 0.21	0.14 ± 0.12	0.29 ± 0.17	↑
*Coprobacillus*	0.01 ± 0.01	0.12 ± 0.36	0.01 ± 0.02	0.10 ± 0.29	↑
*L7A_E11*	0.04 ± 0.04	0.10 ± 0.08	0.15 ± 0.16	0.44 ± 0.76	↑
*Enterococcus*	0.01 ± 0.01	0.05 ± 0.14	0.06 ± 0.32	0.43 ± 0.68	↑
*Escherichia*	0.29 ± 0.26	1.10 ± 2.24	0.24 ± 0.39	2.13 ± 1.41	↑
*Faecalibacterium*	0.06 ± 0.06	0.11 ± 0.13	0.09 ± 0.11	0.14 ± 0.17	↑
*Coprococcus*	1.19 ± 0.61	1.65 ± 1.20	0.90 ± 0.60	1.43 ± 0.78	↑
*Ruminococcus*	1.26 ± 0.43	0.99 ± 0.44	2.06 ± 0.77	1.36 ± 0.82	↓
*Corynebacterium*	0.13 ± 0.1	0.01 ± 0.02	0.18 ± 0.24	0.02 ± 0.02	↓
*Anaerovibrio*	0.25 ± 0.19	0.05 ± 0.08	0.06 ± 0.05	0.02 ± 0.01	↓
*Epulopiscium*	0.43 ± 0.39	0.06 ± 0.09	0.13 ± 0.16	0.05 ± 0.10	↓
*Phascolarctobacterium*	1.45 ± 0.38	0.75 ± 0.46	0.26 ± 0.26	0.17 ± 0.20	↓
*YRC22*	1.31 ± 1.08	0.59 ± 0.85	0.84 ± 0.92	0.23 ± 0.58	↓
*Bifidobacterium*	0.24 ± 0.42	0.08 ± 0.24	0.96 ± 3.09	0.07 ± 0.11	↓
*Akkermansia*	0.11 ± 0.34	0.02 ± 0.06	0.07 ± 0.11	0.01 ± 0.01	↓
*Fibrobacter*	0.52 ± 0.50	0.41 ± 0.70	0.30± 0.62	0.02 ± 0.02	↓
*Prevotella*	10.27 ± 4.56	4.57 ± 2.89	1.57 ± 1.83	0.94 ± 0.66	↓

### Comparison of the Diversity and Composition of Fecal Microbiota in LP, PO, and Barren States

**Figure 3:**
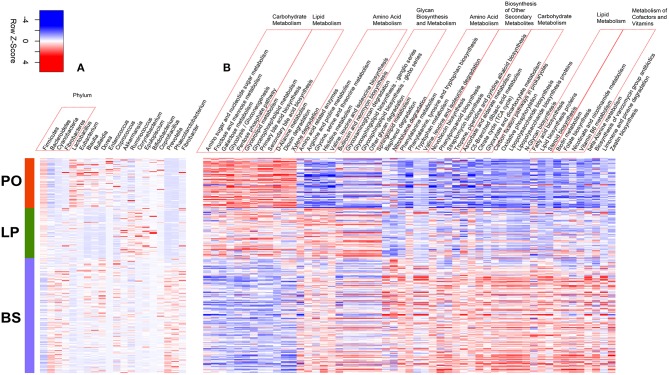
Heatmap of the significant shifts of gut microbial taxa and predicted KEGG pathways from non-pregnancy, later pregnancy to postpartum state. **(A)** The bacterial taxa significantly shifted from non-pregnancy (BS), later pregnancy (LP) to postpartum (PO) state. **(B)** The KEGG pathways showing different abundances from BS, LP to PO state.

To further assess the gut microbiome shift caused by pregnancy and parturition, we compared the microbial diversity and composition in feces among LP, PO, and barren sows (BS) in the F_6_ population, in which fecal samples from 150 non-pregnant F_6_ sows of the heterozygote population were collected and assessed for microbial community structures using 16S rRNA sequencing. Principal Coordinate analysis (PCoA) of the UniFrac Matrix revealed a complete separation amongst the sample sets at the LP, PO, and BS states (Figure 2), suggesting distinct gut microbial community structures between barren sows and sows at the LP and PO state. We further compared the relative abundance of bacterial taxa at the phylum and genus level amongst BS, LP, and PO stages. At the phylum level, we identified four phyla of significantly different abundances at the three reproduction stages (corrected *P* < 0.05). The relative abundance of *Firmicutes* increased from BS, LP to PO, whilst the relative abundance of *Bacteroidetes, Cyanobacteria*, and *Fibrobacteres* significantly decreased (Figure 3A). At the genus level, 16 genera had significantly different abundance levels amongst BS, LP, and PO states. The relative abundance of *Lactobacillus* (one of the predominant genera) significantly increased from 3.38 ± 3.27% (BS), 6.69 ± 5.96% (LP) to 17.99 ± 11.99% (PO). However, the relative abundance of *Prevotella* (another predominant genus) decreased from 12.82 ± 7.58% (BS), 1.59 ± 1.8% (LP) to 1.53 ± 0.98% (PO). The predominant genus *Ruminococcus* showed the highest abundance at LP (BS: 1.65 ± 0.63%; LP: 2.31 ± 0.89%; PO: 1.55 ± 1.16%) (Figure 3A).

### Potential Function Capacity Shifts of the Gut Microbiome From Late Pregnancy to Postpartum

To characterize the potential functional capacity of the gut microbiome and reveal functional shifts from LP to PO, we used PICRUSt to infer the function capacity of the 16S rRNA data. Membrane transport, carbohydrate metabolism and amino acid metabolism were the three most abundant function terms, which occupied about more than 32.0% (32.3 and 32.4%) of the total relative abundance in both F_5_ and F_6_ sows. We next compared the relative abundances of KEGG pathways between sows at LP and PO states, identifying a total of 83 and 74 differential KEGG pathways in the F_5_ and F_6_ sows, respectively. Of these pathways, 68 differential KEGG pathways were present in both F_5_ and F_6_ sows (Table S3). Among these 68 pathways, the relative abundances of 19 pathways were significantly enriched in the gut microbiome at the PO state, including functional terms related to carbohydrate metabolism (fructose and mannose metabolism, amino sugar and nucleotide sugar metabolism, galactose and propanoate metabolism, and the pentose phosphate pathway), lipid metabolism (synthesis and degradation of ketone bodies, primary bile acid and second bile acid synthesis, glycerolipid, and glycerophospholipid metabolism) as well as xenobiotic biodegradation and metabolism. However, the relative abundance of the other 49 pathways were enriched at the LP state, including amino acid metabolism (metabolism of cysteine, methionine, histidine, tryptophan, arginine, and proline), the biosynthesis of other secondary metabolites (butirosin, neomycin, and isoflavonoid biosynthesis), the metabolism of cofactors and vitamins (folate biosynthesis, nicotinate, and nicotinamide metabolism, one carbon pool by folate and vitamin B6 metabolism) in addition to glycan biosynthesis and metabolism. The citrate cycle (TCA cycle) was also enriched at the LP stage (*P* <4.02 × 10^−7^).

We further compared the potential functional capacities of the gut microbiome amongst BS, LP, and PO in F_6_ sows, and identified a total of 53 KEGG pathways of significantly altered abundance amongst the three states. Of these, 12 KEGG pathways including five and four pathways belonging to carbohydrate and lipid metabolism, respectively, successively increased their relative abundance from barren, LP to PO. A total of 11 KEGG pathways were enriched at the LP stage, including five pathways related to amino acid metabolism (branch amino acid biosynthesis, amino acid related enzymes) and four pathways associated with glycan biosynthesis and metabolism. The relative abundance of the other 30 pathways were successively decreased from barren, pregnancy to parturition (Figure 3B).

### Distinct Serum Metabolome Between Sows at Late Pregnancy and Postpartum State

**Figure 4:**
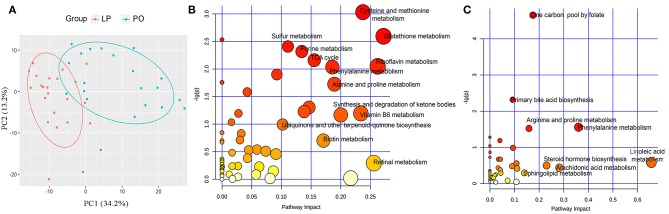
Changes of host serum metabolome, and functional enrichment of differential serum metabolite features. **(A)** PCA analysis indicates the significant changes of host serum metabolome from later pregnancy (LP) to postpartum (PO) state. KEGG pathways enriched by differential metabolite features at LP **(B)** and PO state **(C)**. The *X*-axis and the size of dots indicate the pathway impact of differential metabolite features (the sum of the importance measures of the matched metabolites normalized by the sum of the importance measures of all metabolites in each pathway), and the Y-axis shows the significant *P*-value obtained in enrichment analysis. The size and color of dots indicate the value of pathway impact.

**Figure 5:**
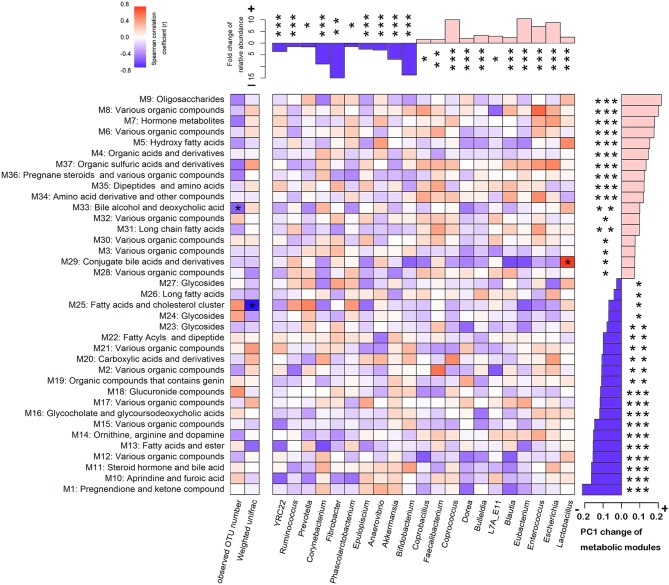
Association between the shifts of gut microbiome and the changes of serum metabolite modules from later pregnancy (LP) to postpartum (PO) state. The bar plot above the heatmap shows the fold change of the relative abundances of microbial taxa. The bar plot on the right side of the heatmap shows the PC1 shifts of metabolic modules. The blue bars represent the enrichment at the LP state, and the pink bars indicate the enrichment at the PO state. The star number indicates the significant threshold *FDR < 0.05, **FDR < 0.01, and ***FDR < 0.001. The different colors of grids show the spearman correlation coefficiency between the changes of microbial taxa and the shifts of host serum metabolome. The stars in the grids indicate the significance.

Serum samples were harvested at both LP and PO stages from each of the 22 F_6_ sows with 16S rRNA gene sequencing data (44 serum samples). Untargeted metabolite profiles were measured to determine the serum metabolome shifts from LP to PO. After quality control, we identified 3,378 quantifiable serum metabolites for further analysis, including 2,110 metabolites from the positive ion mode and 1,268 from the negative ion mode. We initially performed a PCA analysis to evaluate global shifts in the serum metabolome from LP to PO. A separation between the metabolic samples in the LP and PO groups was observed (Figure 4A), suggesting changes in host serum metabolite profiles from LP to PO. Based on the frequent intercorrelation between serum metabolite features (Krumsiek et al., 2011), quantified serum metabolites were clustered into 112 metabolite modules across all individuals using WGCNA methods (Langfelder and Horvath, 2008). We identified 37 of the 112 metabolite modules with significant shifts (the first principal component) from LP to PO (Table S4). Amongst the 37 metabolite modules, 20 were comprised of 639 metabolite features with significantly higher abundances at the LP stage. The other 17 modules consisted of 611 metabolite features with significantly higher abundances at the PO stage (Figure 5). All metabolite features in the modules enriched at the LP stage were performed using KEGG pathway analysis. The same analysis was performed for the metabolites in the modules enriched at the PO state. We found that the metabolites were of higher abundance at the LP stage and enriched in the KEGG pathways of amino acid metabolism (including the metabolism of cysteine, methionine, glutathione, phenylalanine, alanine, and proline), sulfur metabolism, the TCA cycle, and vitamin metabolism (metabolism of riboflavin, vitamin B6, biotin, and retinal). However, the metabolites of higher abundance at the PO stage were enriched in pathways related to lipid metabolism, including primary bile acid biosynthesis, and the metabolism of linoleic acid, arachidonic and sphingolipids (Figures 4B,C). These data were in agreement with the differential KEGG pathways of the gut microbiome between the LP and PO stages.

### Correlation Between the Shift of Gut Microbiota and the Change of Host Serum Metabolites From the LP to the PO State

**Figure 6:**
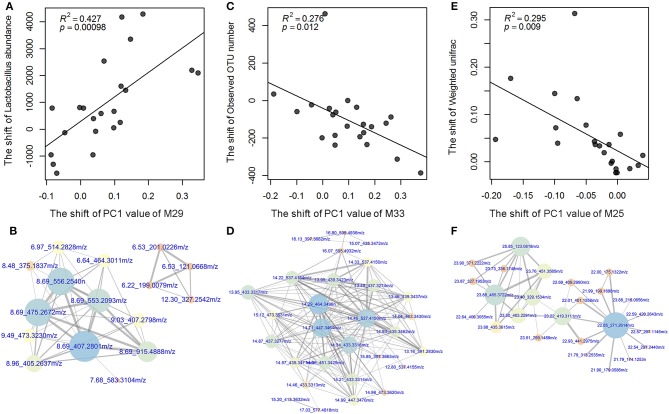
The significant associations between the shifts of *Lactobacillus* abundances and bacterial diversity, and the changes of metabolic modules. **(A)** The shift of the abundance of *Lactobacillus* (the number of read sequences) was positively associated with the change of metabolic module 29 (M29) which was related to bile acid biosynthesis **(B)**. **(C)** The shift of the observed OTU number was negatively associated with the change of metabolic module 33 (M33) which was related to bile acid metabolism **(D)**. **(E)** The shift of the β-diversity of gut microbiota (weighted UniFrac distance) was negatively associated with the change of metabolic module 25 (M25) which was related to lipid metabolism **(F)**. The *X*-axis shows the changed values of host metabolic modules, and the *Y*-axis indicates the changed values of gut microbiota. The size and color of dots indicate the degree of connectivity of hubs, and the thickness of lines shows the strength of correlations. The annotations of metabolite features (m/z) are listed in Table S5.

As described, we identified the distinct α- and β-diversity of the gut microbiota from LP to PO, and 21 bacterial genera and 37 metabolic modules showed significant changes in relative abundance between the two reproduction states. To examine the contribution of the shift of gut microbiota to the changes in host serum metabolites, we performed a correlation analysis in 22 F_6_ sows described above, which had both 16S rRNA gene sequencing and serum metabolome data. As shown in Figure 5, at an FDR < 0.05, we identified three significant correlations between the shift in gut microbiota and changes in the host serum metabolome. The increased abundance of *Lactobacillus* accounted for the observed shift in metabolic module 29 (M29) reflected in the PC1 from the LP to the PO state (Figure 6A). M29 was comprised of 17 serum metabolite features, seven of which were related to cholic acid and conjugated-cholic acid metabolism (Table S5). We further constructed the interaction network for all metabolite features in the M29 and found that cholic acid (8.69_407.2801 m/z) had the biggest hub in the network, suggesting a significant effect of the increased abundance of *Lactobacillus* at the PO state on bile acid biosynthesis (Figure 6B). The second significant correlation was identified between the observed OTU number and the shift of M33 reflected in PC1 from the LP to PO state (Figure 6C). In total, 17 out of the 30 metabolites in M33 were matched to metabolites related to bile acid metabolism (Table S5). The interaction network for the metabolites in the M33 indicated four nodes including Trihydroxycoprostanoic acid (14.29_464.3496 m/z), 2-Deoxycastasterone (14.71_447.3464 m/z), Germanicol cinnamate (14.46_537.4150 m/z), and 3a,7a-Dihydroxycoprostanic acid (14.34_433.3316 m/z), which maintained the strongest lineage with other nodes (Figure 6D). These results suggested that a significant correlation exists between gut microbial richness and bile acid metabolism. We further identified a negative correlation between the β-diversity of gut microbiota (Weighted UniFrac) and the shift of M2M5 (PC1) from LP to PO (*P* = 9.6 × 10^−4^) (Figure 6E). As M25 contained 347 metabolite features constructed through a complex network, we extracted only those nodes ranked in the top 30 of the M25 connectivity network to construct a sub-network. We found that heptadecanoic acid (22.85_271.2614 m/z) and 4,4-Dimethyl-14alpha-formyl-5alpha-cholesta-8-en-3beta-ol (23.88_465.3722 m/z) were the hubs in the sub-network. Heptadecanoic acid (22.85_271.2614m/z) was connected to an array of long-chain fatty acids and their ramifications, including (±)-3-hydroxynonanoic acid, heptadecanoic acid and hexadecyl ferulate. Furthermore, 4,4-Dimethyl-14alpha-formyl-5alpha-cholesta-8-en-3beta-ol (23.88_465.3722m/z) was connected to several metabolites involved in steroid biosynthesis (Figure 6F), suggesting a significant effect of the β-diversity of gut microbiota on host lipid metabolism at the PO state. These results were consistent with shifts in the potential functional capacity of the gut microbiome from LP to PO states, in which the functional terms related to carbohydrate metabolism and lipid metabolism were significantly enriched at the PO stage.

## Discussion

Host metabolism, hormones, and immunity significantly vary during the perinatal period. Whether these changes influence gut microbial composition, and if host-microbe interactions occur that influence host metabolism during this period are largely unknown. Here, we report the dramatic remodeling of gut microbiota and its potential functional capacity from late pregnancy (5 days before parturition) to postpartum (within 6 h after delivery). We observed significant shifts in the host serum metabolome from pregnancy to the postpartum state. These contributed to changes in the host serum metabolites related to lipid and carbohydrate metabolism. Although changes in the gut microbiome in women during pregnancy have been reported (Santacruz et al., 2010; Koren et al., 2012), to our knowledge, this was the first study in pigs reporting alterations of the gut microbiome and host serum metabolic profiles from the last week of pregnancy to the time after delivery, using samples harvested from the same sows. Remodeling of the gut microbiome during the perinatal period was confirmed in two sow populations (F_5_ and F_6_ sows from the same heterogeneous pig cross).

The richness of bacteria in the gut of both pregnant and delivery sows was significantly reduced, whilst the β-diversity dramatically expanded. This contrasted previous studies that reported no significant changes in the relative abundance of *Bacteroidetes* and *Firmicutes* during pregnancy in women (Koren et al., 2012). Similar to the findings in the gut microbial composition reported in obesity (Ley et al., 2006; Turnbaugh et al., 2006), we observed a significant increase in the relative abundance of *Firmicutes*, but reduced levels of *Bacteroidetes* in sows from LP to PO. In women, the relative abundances of *Proteobacteria* and *Actinobacteria* increased on average from the first to the third trimester of pregnancy (Koren et al., 2012). In this study, the increased abundance of *Proteobacteria* was only observed at the postpartum state of F_6_ sows. However, the relative abundance of *Actinobacteria* decreased from LP to PO in F_5_ sows. The relative abundance of *Lactobacillus* significantly increased from LP to PO. Many species of *Lactobacillus* are probiotics used to promote human health, including *Lactobacillus reuteri*, which was shown to upregulate hormone oxytocin and systemic immune responses to achieve a wide array of health benefits, including mental health, metabolism, and myoskeletal maintenance (Erdman and Poutahidis, 2016). It is known that oxytocin induces uterine contraction to facilitate labor in mammals. Oxytocin also plays an important role in the regulation of energy intake during pregnancy (Douglas et al., 2007) and has anti-obesity effects in diet-induced obese rats (Deblon et al., 2011). Furthermore, *Lactobacillus* is related to lipid metabolism through its roles in bile salt biotransformation (Ridlon et al., 2006). The abundance of *Prevotella* significantly decreased from LP to PO. *Prevotella* is a predominant bacterial genus in the gut microbiota, some species of which regulate the uptake and metabolism of peptides and amino acids (Ling and Armstead, 1995; Dai et al., 2011). Consistent with the findings in pregnant women (Koren et al., 2012), the relative abundances of the SCFA-produced bacteria, including *Eubacterium, Coprococcus*, and *Faecalibacterium* were significantly reduced from the barren to LP state (Figure 3A). SCFAs play an important role in anti-inflammatory and restrain obesity (Schwiertz et al., 2010). Low-grade inflammation has been suggested to occur during pregnancy at the intestinal mucosal epithelium (Koren et al., 2012). However, the relative abundance of these bacteria significantly increased from the LP to PO stage, suggesting a change of immune environment after delivery. Studies in humans suggest that a switch from an anti-inflammatory to a pro-inflammatory environment is indispensable for labor once the fetus has completed its development (Mor et al., 2017).

Changes in the potential functional capacity of the gut microbiome were concordance with significant shifts in microbial taxa (Table S3). As described, bacteria related to amino acid metabolism, including *Bacteroidetes, Prevotella*, and *Anaerovibrio*, decreased in abundance from the LP to PO state (Dai et al., 2010). Compared to the sows at BS and PO, sows at the LP stage had a significantly higher abundance of biosynthesis/degradation of valine, leucine, and isoleucine in the gut microbiome. Branched chain amino acids are associated with insulin resistance (Pedersen et al., 2016). In humans, insulin resistance is observed during pregnancy (Barbour et al., 2007). *Prevotella copri* and *Bacteroides vulgatus* are the major species driving the association between the biosynthesis of BCAAs and insulin resistance (Pedersen et al., 2016). *Bifidobacterium* can *de novo* synthesize and supply vitamins. Lactic acid bacteria and *Bifidobacterium* can promote the biosynthesis of B group vitamins (such as folate and riboflavin), and even the complex vitamin B12 (LeBlanc et al., 2013). In this study, the lower abundance of *Bifidobacterium* would be anticipated to decrease the metabolism of cofactors and vitamins from the LP to the PO state. Carbohydrate and lipid metabolism pathways were significantly enriched in sows at the PO state. Drastic metabolic adjustments take place in sows from late pregnancy to lactation to support fetal growth and milk synthesis (Robinson, 1986; Noblet et al., 1990; Butte et al., 1999). The utilization of carbohydrate and lipids continuously increases from late pregnancy to postpartum to support milk synthesis (Butte et al., 1999). Consistent with this, we observed a significant increase in the relative abundance of *Firmicutes* and the ratio of *Firmicutes* to *Bacteroidetes*, which facilitates carbohydrate metabolism. Furthermore, the relative abundance of *Lactobacillus* and SCFA-produced bacteria significantly increased at the PO state. *Lactobacillus* promotes lipid metabolism through its role in bile salt biotransformation (Ridlon et al., 2006).

Host serum metabolome analysis identified differential metabolites from LP to the PO state. Interestingly, these metabolites were enriched in pathways matched to the functional capacity of the gut microbiome, including amino acid metabolism, lipid metabolism and vitamin metabolism, suggesting a relationship between shifts in the gut microbiome, and the host serum metabolome. This relationship was confirmed by correlation analysis from LP to PO. Both the potential functional capacity of the gut microbiome and the metabolites related to amino acid and vitamin metabolism were enriched at the LP stage. As the fetus grows quickly during the LP stage, these changes are likely to support fetal growth. Similarly, the enrichment of the gut microbiome and metabolites related to lipid metabolism at the PO stage should be advantageous to host milk synthesis and lactation, due to the large energy consumption associated with milk production. We identified a significant association of *Lactobacillus* and OTU number for bile acid metabolites. Bile acids increase in abundance during pregnancy (Lunzer et al., 1986) and have been reported to inhibit intestinal anaerobic organisms through direct antimicrobial effects and/or via the induction of antimicrobial peptides. For example, the inhibition of *Bacteroides* and *Clostridia* growth are known to be susceptible to unconjugated bile acids (Binder et al., 1975). This can explain the decreased OTU number of gut microbiota from the LP to PO state, and the negative correlation between OTU number and bile acid metabolites. *Lactobacillus* possesses bile salt hydrolase (BSH) activity (Kumar et al., 2012) which mediates a conserved microbial adaptation to the gut environment and bile tolerance *in vitro*, and enhances bacterial survival in the murine gut *in vivo* (Jones et al., 2008). This may explain the positive association of *Lactobacillus* with bile acid metabolites. These results also provide evidence that bile acids play an important role in mediating host-gut microbiome interactions and shape the gut microbiome and host metabolism during the perinatal period. A significant correlation between the β-diversity of the gut microbiota and metabolites related to fatty acid and cholesterol synthesis was also evident in this study. Cholesterol is a known precursor of bile acids, vitamin D and steroid hormone biosynthesis, all of which are established factors that shape the gut microbiome (Islam et al., 2011; Markle et al., 2013).

In summary, we found a significant alteration of sow gut microbiota and bacterial functional capacities from the final week of pregnancy to postpartum. Amino acid metabolism, glycan biosynthesis and metabolism, and the metabolism of cofactors and vitamins decreased from LP to PO, but bacteria associated with carbohydrate and lipid metabolism within the gut microbiome significantly increased. The host serum metabolome also significantly changed. We identified a significant correlation between the alterations of in the gut microbiome and changes in host serum metabolite features. The results from this study indicate that host-microbial interactions during the perinatal period impact host metabolism, leading to beneficial host changes from pregnancy to delivery and lactation. However, as the limitations, the correlation between the shifts of gut microbiome and the changes of host serum metabolites from LP to PO state was only established based on the association study. The causality and the underlying mechanisms have not been elucidated. These questions will need to be answered in the future study.

## Data Availability

The datasets generated for this study can be found in the SRA database in NCBI with the accession number PRJNA518709.

## Ethics Statement

The animal study was reviewed and approved by the Ministry of Agriculture of China and the Animal Care and Use Committee (ACUC) in Jiangxi Agricultural University.

## Author Contributions

LH designed research, and revised the manuscript. CC designed research, wrote, and revised the manuscript. XH analyzed data and wrote the manuscript. JG performed the experiments and revised the manuscript. YZha analyzed data and performed the experiments. MH, SK, JW, YZho, HF, and HY performed the experiments. All authors read and approved the final manuscript.

### Conflict of Interest Statement

The authors declare that the research was conducted in the absence of any commercial or financial relationships that could be construed as a potential conflict of interest.
